# Effects of Mobilization within 72 h of ICU Admission in Critically Ill Patients: An Updated Systematic Review and Meta-Analysis of Randomized Controlled Trials

**DOI:** 10.3390/jcm12185888

**Published:** 2023-09-11

**Authors:** Ayaka Matsuoka, Shodai Yoshihiro, Haruka Shida, Gen Aikawa, Yoshihisa Fujinami, Yusuke Kawamura, Nobuto Nakanishi, Motohiro Shimizu, Shinichi Watanabe, Kensuke Sugimoto, Shunsuke Taito, Shigeaki Inoue

**Affiliations:** 1 Department of Emergency and Critical Care Medicine, Saga University, 5-1-1 Nabeshima, Saga City 849-8501, Japan; haraherianpan@gmail.com; 2 Department of Pharmacy, Onomichi General Hospital, 1-10-23 Hirahara, Onomichi 722-8508, Japan; shodaidotnet@gmail.com; 3 Scientific Research Works Peer Support Group (SRWS-PSG), Osaka 541-0043, Japan; shutaitou@hiroshima-u.ac.jp; 4 Office of Medical Informatics and Epidemiology, Pharmaceuticals and Medical Devices Agency, Shin-Kasumigaseki Building, 3-3-2 Kasumigaseki, Chiyodaku, Tokyo 100-0013, Japan; haruka2615@gmail.com; 5 Department of Adult Health Nursing, College of Nursing, Ibaraki Christian University, 6-11-1 Omika, Hitachi 319-1295, Japan; gen-aikawa@umin.ac.jp; 6 Department of Emergency Medicine, Kakogawa Central City Hospital, 439 Kakogawacho Honmachi, Kakogawa 675-8611, Japan; greatyoppie@yahoo.co.jp; 7 Department of Rehabilitation, Showa General Hospital, 8-1-1 Hanakoganei, Tokyo 187-8510, Japan; kawa.sgh@gmail.com; 8 Department of Disaster and Emergency Medicine, Kobe University, 7-5-2 Kusunoki, Chuo-ward, Kobe 650-0017, Japan; nobuto_nakanishi@yahoo.co.jp; 9 Department of Intensive Care Medicine, Ryokusen-kai Yonemori Hospital, 1-7-1, Yojiro, Kagoshima 890-0062, Japan; mshimizu@yonemorihp.jp; 10 Department of Physical Therapy, Faculty of Rehabilitation, Gifu University of Health Science, 2-92, Gifu 500-8281, Japan; s-watanabe@gifuhoken.ac.jp; 11 Intensive Care Unit, Gunma University Hospital; 39-15 Showa, Maebashi 371-8511, Japan; k.sugimoto1003@gmail.com; 12 Division of Rehabilitation, Department of Clinical Practice and Support, Hiroshima University Hospital, 1-2-3, Kasumi, Hiroshima 734-8551, Japan

**Keywords:** intensive care unit, post-intensive care syndrome, muscle strength, cognitive function, mental health, quality of life, activities of daily living

## Abstract

Previous systematic review and meta-analysis indicates that rehabilitation within a week of intensive care unit (ICU) admission benefits physical function in critically ill patients. This updated systematic review and meta-analysis aim to clarify effects of initiating rehabilitation within 72 h of ICU admission on long-term physical, cognitive, and mental health. We systematically searched the MEDLINE, Cochrane Central Register of Controlled Trials, and Igaku Chuo Zasshi for randomized controlled trials (RCTs) between April 2019 and November 2022 to add to the previous review. Two investigators independently selected and extracted data. Pooled effect estimates for muscle strength, cognitive function, mental health after discharge, and adverse events were calculated. Evidence certainty was assessed via Grading of Recommendations, Assessment, Development, and Evaluations. Eleven RCTs were included in the meta-analysis. Early rehabilitation may improve muscle strength (three trials; standard mean difference [SMD], 0.16; 95% confidence interval [CI], −0.04–0.36) and cognitive function (two trials; SMD, 0.54; 95% CI, −0.13–1.20). Contrastingly, early mobilization showed limited impact on mental health or adverse events. In summary, initiating rehabilitation for critically ill patients within 72 h may improve physical and cognitive function to prevent post-intensive care syndrome without increasing adverse events. The effect on mental function remains uncertain.

## 1. Introduction

Post-intensive care syndrome (PICS) is a new or worsening physical, cognitive, or mental health disorder that occurs and persists after hospitalization for a critical illness, leading to a decreased quality of life (QOL) [1,2]. Its development is significantly linked to increased post-discharge mortality [3]. In recent years, the number of individuals with PICS has increased as more patients survive and are discharged from the intensive care unit (ICU), owing to improved treatment and care. It has been reported that approximately 50% of critically ill patients experience at least one PICS-related symptom [4]. Therefore, interventions to prevent PICS are important in ICU care [1,5,6].

Previous systematic review and meta-analysis (SRMA) has demonstrated that initiating rehabilitation within one week of ICU admission among critically ill patients correlates with enhanced Medical Research Council (MRC) scale scores and a reduced incidence of muscle weakness during ICU stays. Essentially, these findings suggest that commencing rehabilitation within the first week of ICU admission primarily impacts the physical function aspect of PICS [7,8]. However, uncertainty surrounds the influence of early rehabilitation on cognitive and mental function, as well as on overall QOL, as indicated by this SRMA.

Recent reports have shown that physical function improvement can be expected in some patients when rehabilitation is started within 72 h of ICU admission [9]. Nevertheless, there remains a lack of established knowledge regarding the effects of beginning rehabilitation within this 72 h window on the various components of PICS—namely, physical, cognitive, and mental function—as well as limited insight into its safety implications.

Therefore, in this SRMA, we aimed to review randomized controlled trials (RCTs) to examine the effects of rehabilitation initiated within 72 h of ICU admission on the long-term physical, cognitive, and mental functioning of critically ill patients and on the safety of rehabilitation.

## 2. Materials and Methods

### 2.1. PICOTS

The patient population under study comprised critically ill patients within the ICU. The intervention being investigated was early rehabilitation, defined as commencing within 72 h of ICU admission. The intervention involved initiating rehabilitation earlier than in the control group. The control group received usual care. The primary outcomes assessed were muscle strength, cognitive function, and adverse events associated with rehabilitation, serving as indicators of physical function in the context of PICS. Secondary outcomes included activities of daily living (ADL), QOL, mental function, and mortality. The study’s settings encompassed both the ICU and the post-discharge period. As a general guideline, parameters concerning muscle strength, cognitive function, mental function, ADL, and QOL were measured approximately 6 months after discharge. However, in cases where values at the 6-month mark were unavailable, measurements taken closest to the 6-month point post-discharge were used.

### 2.2. Study Registration

This study is an updated SRMA of the Japanese Clinical Practice Guidelines for the Management of Sepsis and Septic Shock 2020 and a previous systematic review [8,10]. The study protocol was registered with the International Prospective Register of Systematic Reviews (PROSPERO, registration number 42022372944). Further, our study protocol and reporting adhered to the Preferred Reporting Items for Systematic Reviews and Meta-analyses (PRISMA) statement (Table S1).

### 2.3. Search Strategy

We searched the following databases for eligible trials: the Cochrane Central Register of Controlled Trials (CENTRAL) in the Cochrane Library, MEDLINE via PubMed, and Igaku Chuo Zasshi. The studies were limited to those published in English or Japanese. We searched for studies conducted between April 2019 and November 2022 because the previous systematic review search was conducted in April 2019 [8] (Table S2). We also reviewed articles cited by the Clinical Practice Guidelines for the Prevention and Management of Pain, Agitation/Sedation, Delirium, Immobility, and Sleep Disruption in Adult Patients in the ICU [11], as well as the reference lists of the included studies and any articles that cite these studies. In the context of the previous meta-analysis conducted by Okada et al., we examined the timing of rehabilitation initiation. Studies in which rehabilitation commenced within 72 h of ICU admission were also incorporated into the present meta-analysis.

### 2.4. Data Extraction

Two independent researchers (AM and HS) performed study selection and exclusion and extracted data using the titles and abstracts. The full text of all selected articles was independently reviewed by the two researchers to determine whether the articles were eligible for final inclusion in the review. Any disagreements between the two reviewers were discussed and resolved; if this failed, a third reviewer (SY) acted as an arbiter.

### 2.5. Eligibility Criteria

Only RCTs involving critically ill adult (≥18 years) patients admitted to the ICU were included in the study. Interventions were defined as early mobilization initiated within 72 h of ICU admission or earlier than that conducted in the control group [12]. Studies using only neuromuscular electrical stimulation or ergometers were excluded. Controls were defined as individuals who received usual care, or mobilization programs initiated after the intervention. Studies in which the distinction between the intervention and control groups revolved around the intensity or frequency of rehabilitation, even when early rehabilitation was implemented, were also excluded.

### 2.6. Outcomes

Muscle strength, cognitive function, and adverse events were assessed as primary outcomes. We used the MRC scores and grip strength as measures of muscle strength, prioritizing the MRC during data extraction if both were measured. We evaluated muscle strength and cognitive function measured 6 months after hospital discharge, or sooner if these factors were not assessed at 6 months. Adverse events included all adverse events identified during each study period. ADL, QOL, mental health, and mortality were secondary outcomes in our study. The Barthel index, a functional independence metric, was used to measure ADL, and the Medical Outcomes Study Short-Form 36-Item Health Survey (SF-36) and EuroQol five-dimension questionnaire (EQ-5D) were used to assess QOL. ADL, QOL, and mental health were measured approximately 6 months after hospital discharge. Finally, mortality was measured 28–30 days after discharge, and hospital mortality was determined if it was not evaluated in the original study.

### 2.7. Risk-Of-Bias Assessment

When evaluating the risk of bias, each study was individually assessed for specific outcomes, focusing on aspects such as the randomization process, deviations from intended interventions, missing outcome data, measurement of the outcome, and selection of the reported results. An overarching assessment was subsequently derived from these various domains. The Cochrane Risk of Bias 2 (RoB 2) tool [13] is used to assess the quality of the study design and the degree of potential bias according to specific domains. Two reviewers (AM and HS) independently evaluated the risk of bias using the RoB 2. Disagreements were discussed, and if they could not be resolved, a third reviewer (SY) acted as an arbiter. 

### 2.8. Data Synthesis Strategy

A meta-analysis was conducted using Review Manager software version 5.4 (Cochrane, London, England) in accordance with the “Cochrane Handbook for Systematic Reviews of Interventions” [14] and the PRISMA statement. The risk ratio (RR) was calculated in the analysis of binary variables. For continuous variables, the mean difference (MD) was determined when integrating the same parameters, whereas the standard mean difference (SMD) was used when integrating different parameters. The certainty of evidence (CoE) was determined by evaluating the following five domains: risk of bias, inconsistency, indirectness, imprecision, and other considerations. For each domain, no downgrade, a one-step downgrade, or a two-step downgrade was assigned. 

### 2.9. “Summary of Findings” Table

The CoE was established through an evaluation of five domains: risk of bias, non-consistency, non-directness, imprecision, and other considerations. A “high” CoE was assigned when no downgrading or one-step downgrading was applied within each domain, with no points being deducted. A CoE of “moderate” was attributed if there was a one-level downgrade, while a “low“ CoE was given if a two-level downgrade was applicable. Furthermore, a CoE of “very low” was designated if the evidence was downgraded by three levels or more. The two reviewers (AM and HS) evaluated the CoE based on the Grading of Recommendations Assessment, Development, and Evaluation (GRADE) approach [15]. Disagreements between the reviewers were discussed, and a third reviewer (SY) acted as an arbiter when no resolution could be reached. A “summary of findings” table was created for all the study outcomes based on the Cochrane handbook.

### 2.10. Evaluation of Heterogeneity

Heterogeneity was evaluated by visual inspection of forest plots and calculation of the I^2^ statistic to quantify the magnitude of statistical heterogeneity among the included studies. Heterogeneity was defined as follows: I^2^ = 0–40%, possibly not important; 30–60%, possible moderate heterogeneity; 50–90%, possible substantial heterogeneity; 75–100%, considerable heterogeneity. If heterogeneity was detected (I^2^ > 50%), we examined its causes using a Cochrane chi-square test (Q-test) performed for the I^2^ value, with *p* < 0.10 considered statistically significant. If significant heterogeneity was identified, the median of the estimates was reported instead of the weighted estimates.

### 2.11. Missing Values 

The original authors were contacted in cases of missing data. If the missing value remained unknown, we performed an intention-to-treat analysis for all dichotomous data, if possible. For continuous data, we imputed missing data based on the recommendations of the Cochrane handbook. We performed a meta-analysis of the data available in the original studies.

### 2.12. Deviations from Protocol

The PROSPERO-registered protocol included subgroup analyses for studies involving patients with sepsis or those in which rehabilitation was performed at least five times per week. However, only one study was conducted in patients with sepsis, and the outcomes required for analysis were not measured. Furthermore, subgroup analyses were not conducted because the interventions were performed at least five times per week in all the included studies.

## 3. Results

### 3.1. Search Results

**Figure 1:**
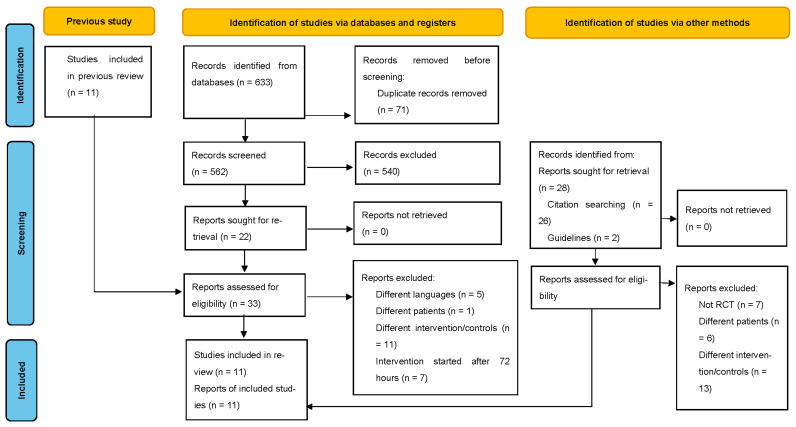
PRISMA flow diagram of study selection. RCT, randomized controlled trial.

The PRISMA flow diagram of the study selection process is shown in Figure 1. The updated search retrieved 631 studies, of which 24 were included in the full-text screening process. Of those assessed in the previous SRMA [8], seven studies described interventions within 72 h of ICU admission [16,17,18,19,20,21,22]. Four studies were newly included in the current SRMA [23,24,25,26]. In total, 11 studies were included in the meta-analysis after excluding those written in languages other than English and Japanese, studies that were not RCTs, those that did not incorporate critically ill patients, those that started the intervention after 72 h, and studies with interventions other than early rehabilitation (Table S3). The characteristics of the 11 studies [16,17,18,19,20,21,22,23,24,25,26] are shown in Table 1. The included RCTs were published between 2009 and 2023. The patient cohort sizes across the studies displayed variation, ranging from 10 patients in both the intervention and comparison groups, to larger trials involving 150 patients in each group. The sizes of the RCTs exhibited diversity. Among the studies, only one—conducted by Kayambu—focused exclusively on septic patients. Several other studies encompassed the entirety of ICU patients, while the majority concentrated on patients requiring mechanical ventilation. Notably, six studies [17,20,21,22,23,24] evaluated primary outcomes, with all interventions being administered on a daily basis throughout these studies.

### 3.2. Meta-Analysis

#### 3.2.1. Primary Outcomes

**Figure 2:**
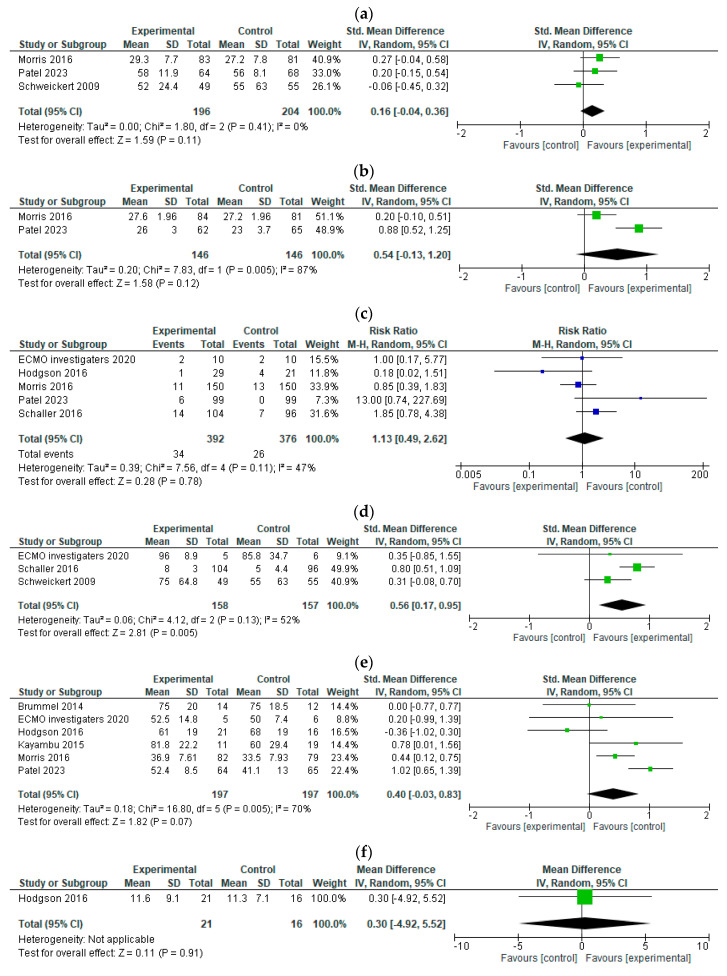

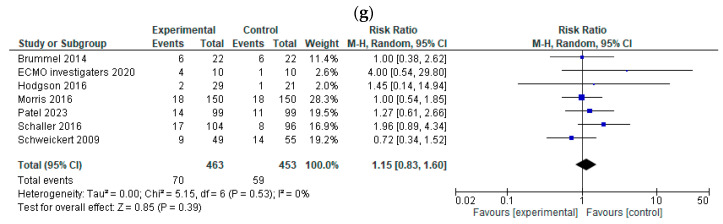
Forest plot of each primary and secondary outcome [16,17,18,20,21,22,23,24]. (**a**) Muscle strength. (**b**) Cognitive function. (**c**) Adverse effects. (**d**) Activities of daily living. (**e**) Quality of life. (**f**) Mental health. (**g**) Mortality. SD, standard deviation; CI, confidence interval; df, degrees of freedom. Blue blocks indicate RR and green blocks indicate MD or SMD.

Muscle strength. Three RCTs [20,22,24] evaluated the effect of early mobilization on muscle strength. One study measured grip strength [20] and two reported MRC scores [22,24]. The meta-analysis showed that early mobilization slightly increased muscle strength (N = 400; SMD, 0.16; 95% CI, −0.04–0.36; CoE, low; Figure 2a, Table 2). The risk of bias was defined as serious due to the presence of some risk in the selection of the reported results (Figure S1a). Additionally, the level of imprecision was rated as serious due to insufficient sample size, and the CoE was reduced to low.

Cognitive function. Two RCTs [20,24] investigated the effects of early mobilization on cognitive function. One study used the Mini-Mental State Examination [20], and the other used the Montreal Cognitive Assessment [24] to assess cognitive function. Our results showed that early mobilization likely improved cognitive functioning (N = 292; SMD, 0.54; 95% CI, −0.13–1.2; CoE, moderate; Figure 2b, Table 2). The risk of bias was not serious (Figure S1b); however, imprecision was rated as serious due to an insufficient sample size, and the CoE was lowered to moderate.

Adverse events. Five RCTs [17,20,21,23,24] evaluated the effects of early mobilization on any adverse events. Adverse events were reported in 34 of the 392 participants in the intervention groups and in 26 of the 376 individuals in the control groups. Morris et al. reported bradycardia as a potentially life-threatening adverse reaction in the intervention group that was thought to be related to the intervention. Other reported adverse events were not life-threatening and included decreased blood pressure, decreased oxygenation, and arterial catheter or nasogastric tube disposition. The meta-analysis suggested that early mobilization did not increase the incidence of adverse events (N = 768; RR, 1.13; 95% CI, 0.49–2.62; CoE, low; Figure 2c, Table 2). 

#### 3.2.2. Secondary Outcomes

Activities of Daily Living. Three RCTs [21,22,23] assessed the effects of early mobilization on ADL. Two studies used the Barthel index [22,23] and one study used the Functional Independence Measure [19] to determine ADL. We observed that early mobilization improved ADL (N = 315; SMD, 0.56; 95% CI, 0.17–0.95; CoE, low; Figure 2d, Table 2). The risk of bias was rated as serious due to the presence of some risk in missing outcome data (Figure S1d). Additionally, imprecision was defined as serious due to an insufficient sample size, and the CoE was reduced to low.

Quality of Life. Six RCTs [15,16,17,18,20,23,24] evaluated the effects of early mobilization on QOL. To measure QOL, three studies used the SF-36 [18,20,24], two used the EQ-5D visual analog scale [16,17], and one used the EQ-5D-5L [23]. Early mobilization may have improved QOL (N = 394; SMD, 0.53; 95% CI, 0.32–0.73; CoE, low; Figure 2e, Table 2). The risk of bias was defined as serious due to the presence of some risk in missing outcome data (Figure S1e). Imprecision was also rated to be serious due to an insufficient sample size, and the CoE was reduced to low.

Mental health. One RCT [17] evaluated the effects of early mobilization on mental health using the Hospital Anxiety and Depression Scale. Our results showed that early mobilization resulted in little to no difference in mental health (N = 37; MD, 0.30; 95% CI, −4.92–5.52; CoE, low; Figure 2f, Table 2). The risk of bias was rated as serious due to potential bias in the selection of the reported results (Figure S1f). Furthermore, imprecision was defined as serious due to an insufficient sample size. The CoE was reduced to low.

Mortality. Seven RCTs [16,17,20,21,23,24] evaluated the effects of early mobilization on short-term mortality. We observed that early mobilization resulted in little to no difference in short-term mortality (N = 916; RR, 1.15; 95% CI, 0.83–1.60; CoE, low; Figure 2g, Table 2). The risk of bias was determined not to be serious (Figure S1g). However, imprecision was rated as very serious due to an insufficient sample size and a wide 95% CI. Therefore, the CoE was reduced to low.

## 4. Discussion

We conducted an updated SRMA of 1301 patients in 11 RCTs to investigate the prognostic impact of early rehabilitation in critically ill patients in the ICU. According to the GRADE framework, which is based on point estimates and CoE evaluation, we determined that early rehabilitation of critically ill patients may improve cognitive function. In addition, early mobilization may have ameliorated muscle strength, ADL, and QOL without increasing mortality or adverse events.

Early rehabilitation starting less than 72 h after admission may improve physical and cognitive function and prevent PICS. Fuke et al. performed a systematic review on the impact of early rehabilitation, defined as rehabilitation initiated within 1 week of ICU admission, on PICS in critically ill patients [7]. The authors reported that early rehabilitation only improved short-term physical function in the three PICS domains [7]. Similarly, Okada et al. reported the possibility of improving physical function through early mobilization [8]. Recently, rehabilitation within 72 h of ICU admission has been reported to improve functional prognosis [9]. In the present meta-analysis, we showed that early rehabilitation limited to the first 72 h leads to improvements in both physical and cognitive function, which may result in improvement in the two corresponding PICS domains. However, our systematic review did not reveal any improvement in the mental health induced by early rehabilitation. Of the 11 studies included in the meta-analysis, only one reported mental health as an outcome, and further assessment of this domain will be necessary in the future.

The SRMA showed that early rehabilitation can be performed safely without increasing the incidence of adverse events or death. In a previous systematic review, Nydahl et al. reported that early rehabilitation in critically ill patients was not associated with more adverse events [27]. Another systematic review reported that mortality could also be reduced owing to early rehabilitation [8]. In the RCTs included in our SRMA, no serious adverse events such as endotracheal tube removal, vascular access device removal, falls, or cardiac arrest were reported. Even if the start of rehabilitation is limited to 72 h after ICU admission, our results show that mobilization of critically ill patients can be safely performed. 

The RCT conducted by Hodgson et al. in 2022 [28] was not considered in this SRMA due to the absence of disparity in the timing of rehabilitation initiation between the intervention and control groups. In this study, the intervention group experienced a higher intensity of rehabilitation as compared to the control group, leading to an escalation in rehabilitation-associated adverse events. It is noteworthy that rehabilitation was initiated within 24 h of ICU admission in this study. This instance underscores the potential for increased adverse events associated with early rehabilitation, contingent on the intensity and timing of the initiation of rehabilitation. Further studies are needed to clarify the appropriate dose and frequency of early mobilization to avoid adverse events.

This review suggests that starting rehabilitation within 72 h prevents PICS and improves ADL and QOL. Previous reports state that early rehabilitation improves mobility and ADL [29,30,31], although there is insufficient evidence on its impact on QOL [30,31,32]. Furthermore, previous SRMAs have reported that more frequent rehabilitation may be more effective in improving QOL [31]. Conversely, another SRMA showed that early rehabilitation starting within 1 week of ICU admission did not effectively improve QOL [6]. The present review suggests that early rehabilitation may improve ADL and QOL, which is a new finding that differs from those of previous SRMAs [32,33,34]. Commencing early rehabilitation within 72 h may be more effective in improving ADL and QOL, in addition to preventing PICS.

In this study, the effect of early rehabilitation on mental functioning remained uncertain. Observational studies investigating the long-term physical and mental functional outcomes of COVID-19 critically ill patients one year post-ICU discharge have indicated an association between PTSD and a reduced QOL [35]. Within the scope of the present study, only one RCT encompassed mental function as an outcome measure. Additional research is warranted to delve into the effects of early rehabilitation on mental functioning among critically ill patients, as well as to explore the potential link between mental functioning and QOL.

This study has several limitations. First, the number of studies included in the SRMA was small, and subgroup analyses could not be performed. Second, the timing of outcome measurements differed among the included studies, ranging from 3–12 months after hospital discharge. Third, rehabilitation in the control group was described as “usual care” or “hospital standard treatment” in each article, with no detailed description of what these treatments entailed. In a previous systematic review, early rehabilitation reportedly did not improve physical function when the control group received high-dose as opposed to low-dose rehabilitation [36]. The intensity and frequency of rehabilitation in the control groups in our study was unclear, and variations in the type of rehabilitation performed in the controls might have affected the outcomes.

## 5. Conclusions

Initiating rehabilitation within 72 h of ICU admission in critically ill patients may improve physical and cognitive function, preventing PICS without increasing the rate of adverse events. The effect of early rehabilitation on mental functioning remains unknown based on the findings of the present study. Further large-scale studies with a low risk of bias are needed to clarify the impact of early rehabilitation on mental health.

## Figures and Tables

**Table 1:** Patient characteristics of trials included in the meta-analysis.

Authors	Year	Target Population	Number of Patients	Sex (Male)	Age (Years)	Physiological Severity
			I/C	I/C	I/C	I/C
Schweickert [22]	2009	Sedated adult patients with MV in ICU	49/55	20/22	57.5 (36.3–69.1)/54.4 (46.5–66.4)	20 (15.8–24.0)/19 (13.3–23.0) ^a^
Brummel [16]	2014	Adult patients with respiratory failure and/or shock in ICU	22/22	13/8	62 (48–67)/60 (51–69)	21.5 (20.0–28.8)/27 (17.5–31.0) ^a^
Kayambu [18]	2015	Critically ill adult patients admitted to ICU with sepsis	26/24	18/14	62.5 (30–83)/65.5 (37–85)	28 (7.6)/28 (6.8) ^a^
Morris [20]	2016	Adult patients admitted to ICU with MV	150/150	66/68	55 (17)/58 (14)	76 (26)/75 (27) ^b^
Schaller [21]	2016	Adult patients with MV in SICU	104/96	65/61	66 (48–73)/64 (45–76)	16 (12–22/17 (11–22) ^a^
Hodgson [17]	2016	Critically ill adult patients with MV in ICU	29/21	21/9	64 (12)/53 (9)	43 (14)/45 (12) ^a^
Moradian [19]	2017	Adult patients who underwent CABG	49/49	33/30	59 (10)/60 (11.3)	-/-
Schujmann [25]	2020	Adult patients admitted to ICU	68/67	55/48	48 (15)/55 (12)	51 (9)/52 (9) ^c^
ECMO investigators [23]	2020	Adult patients with ECMO in ICU	10/10	8/8	49.3 (13.4)/50.6 (17.1)	24.4 (5.9)/19.4 (4.8) ^a^
Zhou [26]	2022	Adult patients admitted to ICU for the first time	50/50	26/30	57.0 (15.3)/57.3 (13.7)	13.9 (5.1)/14.0 (6.3) ^a^
Patel [24]	2023	Adult patients with MV in ICU	99/99	58/55	57.9 (42.3–66.8)/54.5 (41.9–64.7)	23 (18–29)/23 (16–27) ^a^

Age and physiological severity are expressed as mean and standard deviation or median and interquartile range. ^a^ APACHE2, ^b^ APACHE3, ^c^ SAPS2. I/C, intervention/control; APACHE2/3: Acute Physiology and Chronic Health Evaluation 2/3 score; SAPS2, Simplified Acute Physiology Score; ICU, intensive care unit; MV, mechanical ventilation; SICU, surgical ICU; CABG, coronary artery bypass graft; ECMO, extracorporeal membrane oxygenation.

**Table 2:** Summary of findings for each study outcome.

Early rehabilitation compared to control for ICU-AW						
Patient or population: Critically ill patients Setting: Intensive care unit Intervention: Early rehabilitation Comparison: Control						
**Outcomes**	Anticipated absolute effects * (95% CI)		**Relative effect** **(95% CI)**	**No. of participants** **(studies)**	**Certainty of the evidence** **(GRADE)**	**Comments**
	**Risk with Control**	**Risk with Early rehabilitation**				
Muscle strength	-	SMD 0.16 higher (0.04 lower to 0.36 higher)	-	400 (3 RCTs)	⨁⨁◯◯ Low ^a,b^	
Cognitive function	-	SMD 0.54 SD higher (1.3 lower to 1.2 higher)	-	292 (2 RCTs)	⨁⨁⨁◯ Moderate ^b^	
Adverse effects	69 per 1000	78 per 1000 (34 to 181)	RR 1.13 (0.49 to 2.62)	768 (5 RCTs)	⨁⨁◯◯ Low ^c^	
ADL	-	SMD 0.56 higher (0.17 higher to 0.95 higher)	-	315 (3 RCTs)	⨁⨁◯◯ Low ^b,d^	
Quality of life	-	SMD 0.53 higher (0.32 higher to 0.73 higher)	-	394 (6 RCTs)	⨁⨁◯◯ Low ^b,d^	
Mental health		MD 0.3 higher (4.92 lower to 5.52 higher)	-	37 (1 RCT)	⨁⨁◯◯ Low ^a,b^	
Mortality	130 per 1000	150 per 1000 (108 to 208)	RR 1.15 (0.83 to 1.60)	916 (7 RCTs)	⨁⨁⨁◯ Moderate ^b^	

* The risk in the intervention group (and its 95% confidence interval) is based on the assumed risk in the comparison group and the relative effect of the intervention (and its 95% CI). ADL, activities of daily living; GRADE, Grading of Recommendations Assessment, Development, and Evaluation; ICU-AW, ICU-acquired weakness; MD, mean difference; RR, risk ratio; SMD, standardized mean difference. Explanations: ^a^ Downgraded one level due to some risk of bias in selection of the reported result; ^b^ Downgraded one level due to insufficient simple size; ^c^ Downgraded two levels due to insufficient sample size and a wide 95% CI; ^d^ Downgraded one level due to high risk of bias in missing outcome data. ⨁: described as Low; ◯: described as Low or Moderate below it.

## Data Availability

All relevant data are within the manuscript and Supplementary Materials.

## Supplementary Materials

The following supporting information can be downloaded at https://www.mdpi.com/article/10.3390/jcm12185888/s1: Table S1. Prisma 2020 Checklist; Table S2. Search strategies; Table S3. Studies excluded in full-text screening; Figure S1. Risk-of-bias assessment for each study outcome.
