# Supplementary material for: Effects of Mobilization within 72 h of ICU Admission in Critically Ill Patients: An Updated Systematic Review and Meta-Analysis of Randomized Controlled Trials
Source: J Clin Med. 2023 Sep 11;12(18):5888. doi: 10.3390/jcm12185888 (PMC10531519; doi:10.3390/jcm12185888)
Supplement: Supplementary file 1 [file jcm-12-05888-s001.zip › Supplementary Figure S1.pdf]

a. Muscle strength

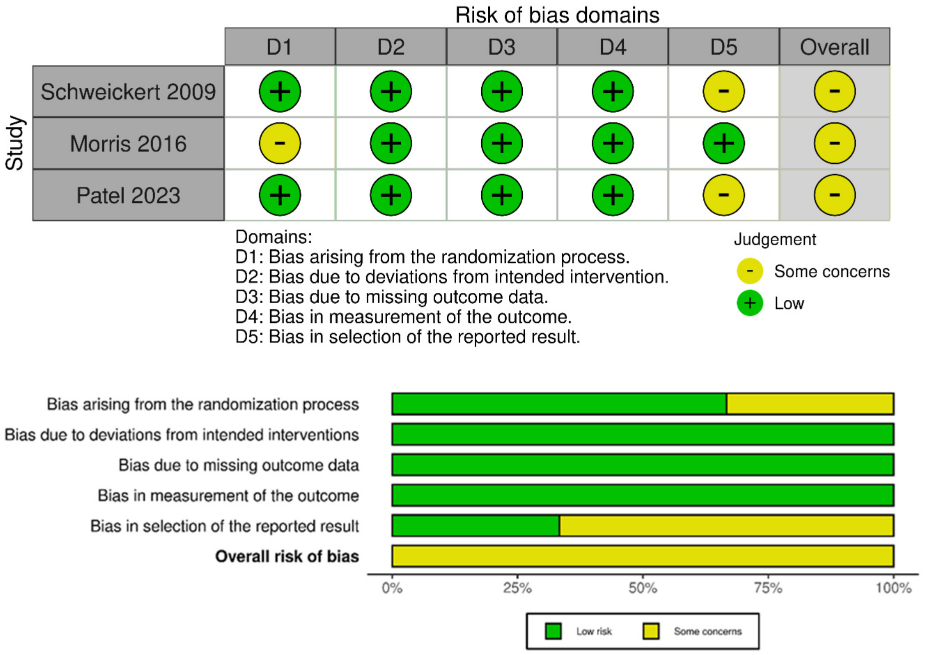

b. Cognitive function

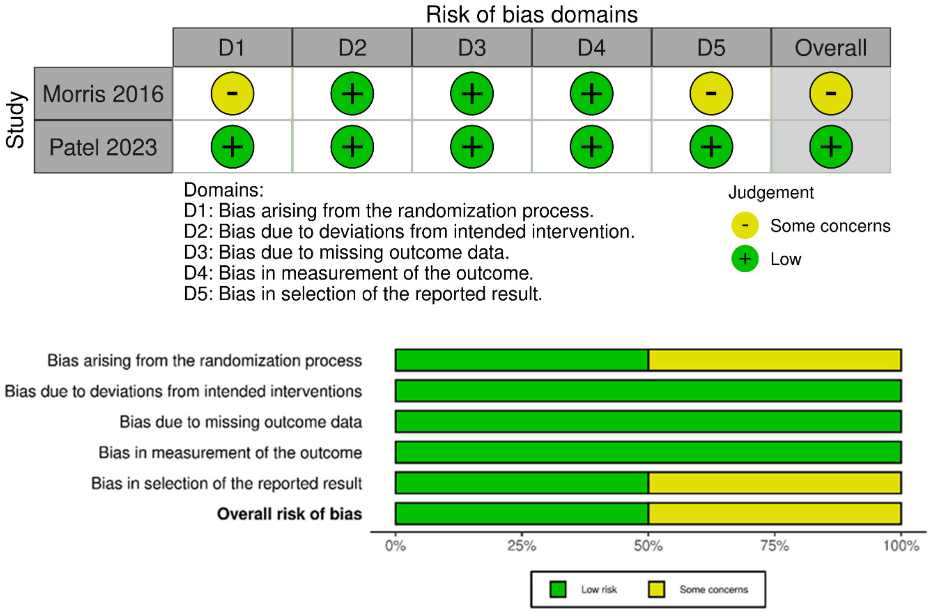

c. All adverse effects

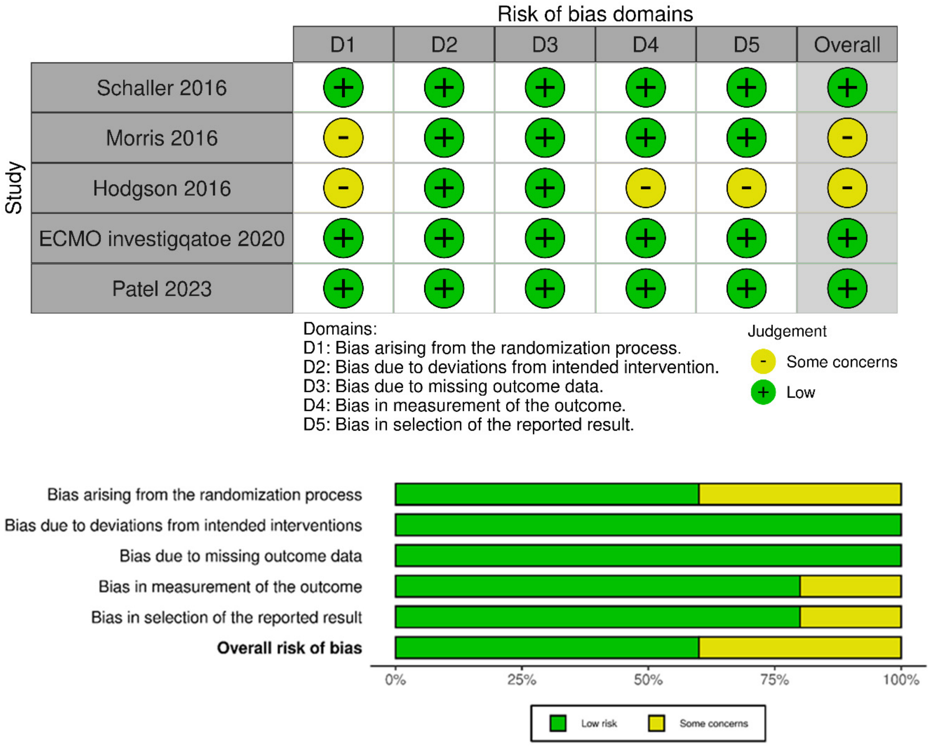

d. Actives of daily living

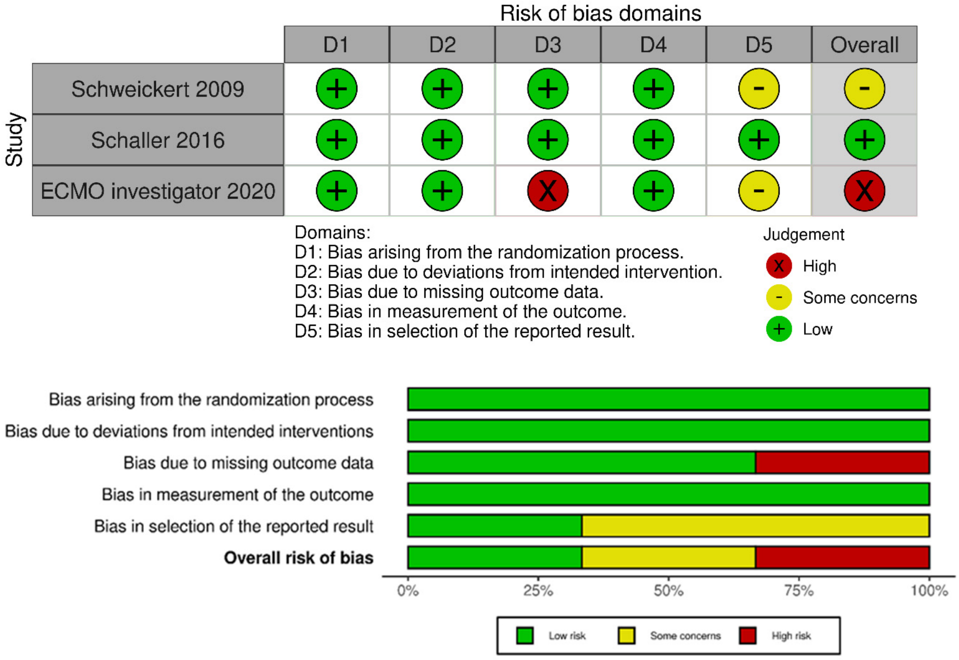

e. Quality of life

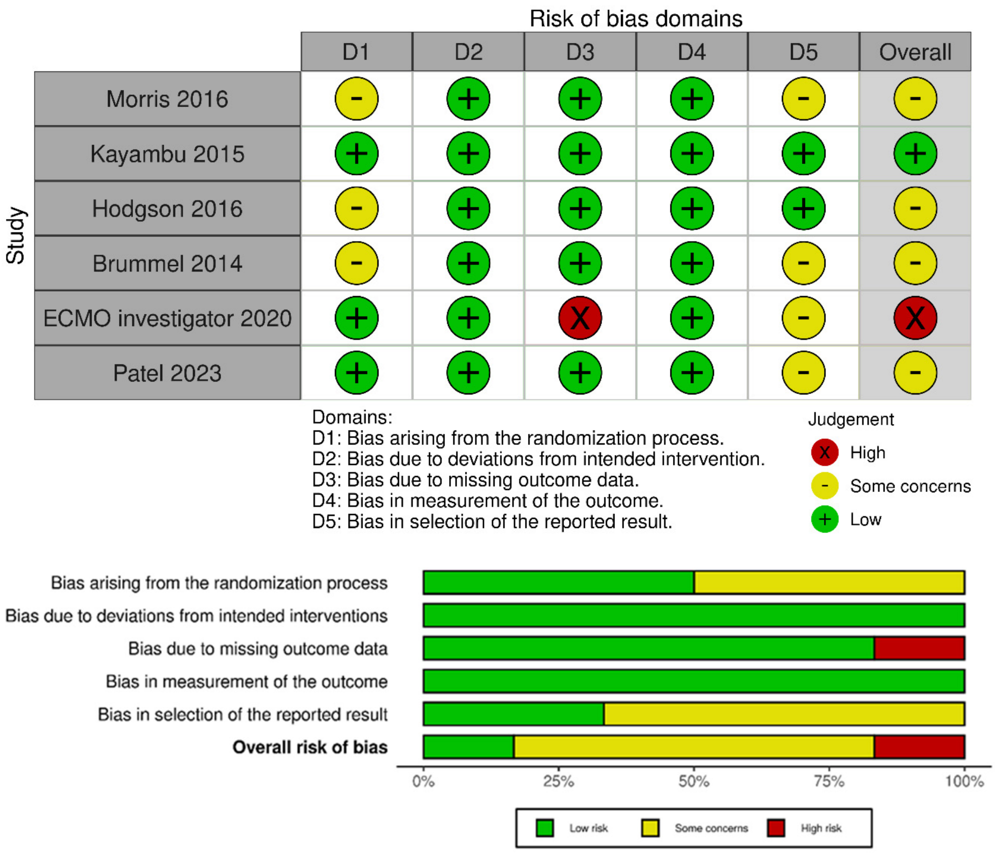

f. Mental health

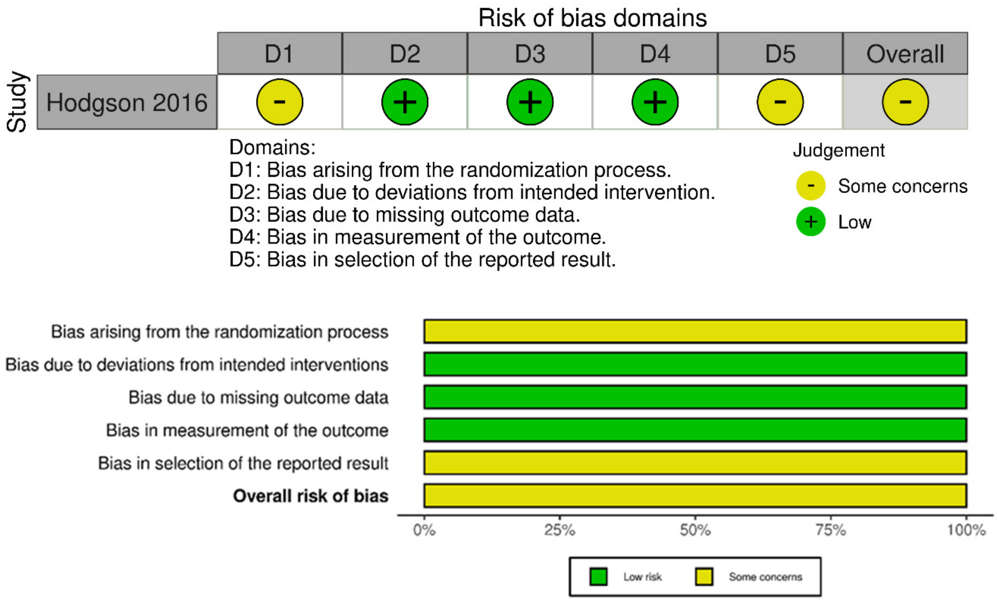

g. Mortality

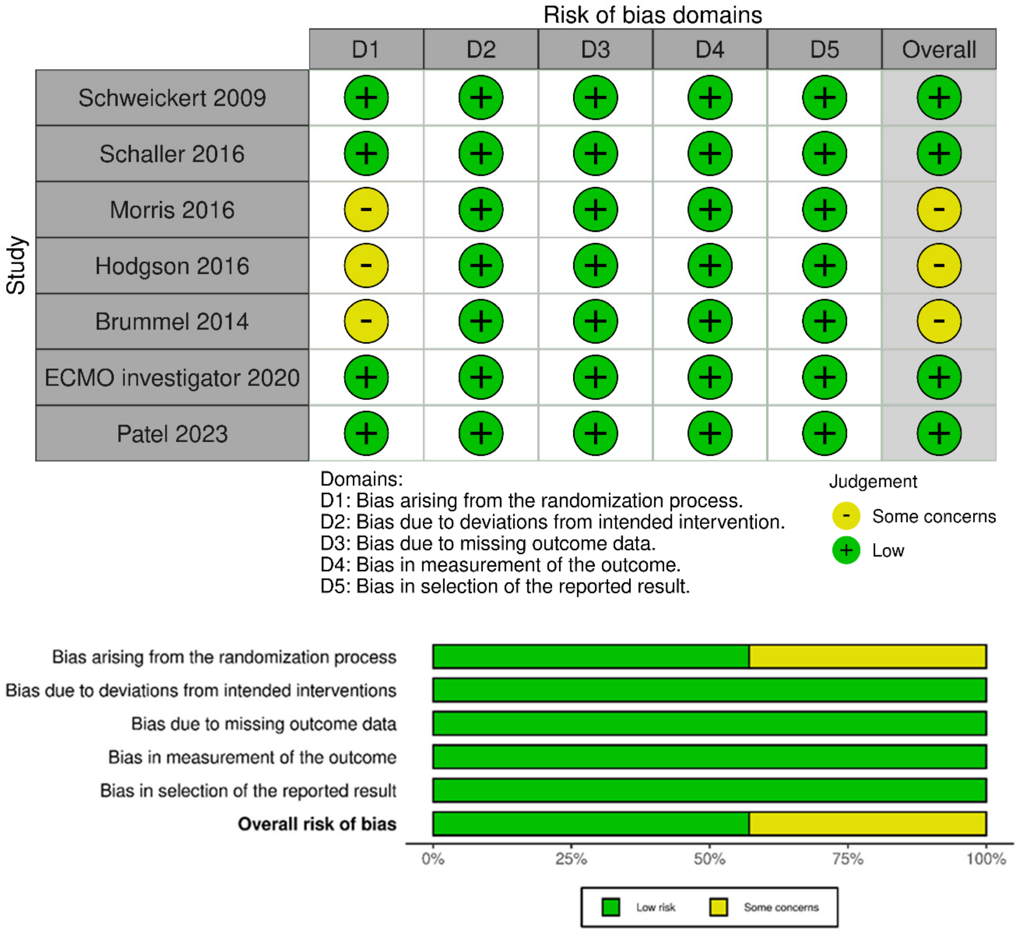

Figure S1. Risk-of-bias assessment for each study outcome. (**a**) Muscle strength. (**b**) Cognitive function. (**c**) All adverse effects. (**d**) Activities of daily living. (**e**) Quality of life. (**f**) Mental health. (**g**) Mortality.
