# Supplementary material for: Effects of Mobilization within 72 h of ICU Admission in Critically Ill Patients: An Updated Systematic Review and Meta-Analysis of Randomized Controlled Trials
Source: J Clin Med. 2023 Sep 11;12(18):5888. doi: 10.3390/jcm12185888 (PMC10531519; doi:10.3390/jcm12185888)
Supplement: Supplementary file 1 [file jcm-12-05888-s001.zip › Supplementary Table S2.pdf]

Table S2. Search strategies

Databases

- MEDLINE
- CENTRAL
- Ichushi-Web

MEDLINE (via PubMed) search strategy（November 8, 2022）

#1	"physical therapy modalities"[MeSH Terms]
#2	"occupational therapy"[MeSH Terms]
#3	"early ambulation"[MeSH Terms]
#4	"mobility"[Title/Abstract]
#5	"training"[Title/Abstract]
#6	"pregait"[Title/Abstract]
#7	"pre-gait"[Title/Abstract]
#8	"ADL"[Title/Abstract]
#9	"ambulation"[Title/Abstract]
#10	mobiliz*[Title/Abstract]
#11	mobilis*[Title/Abstract]
#12	exercis*[Title/Abstract]
#13	physiotherap*[Title/Abstract]
#14	"occupational"[Title/Abstract]
#15	walk*[Title/Abstract]
#16	"physical therapy"[Title/Abstract]
#17	#1 OR #2 OR #3 OR #4 OR #5 OR #6 OR #7 OR #8 OR #9 OR #10 OR #11 OR #12 OR #13 OR #14 OR #15 OR #16
#18	((((("critical illness"[Mesh Terms]) OR "intensive care units"[Mesh Terms]) OR "critical care"[Mesh Terms])) OR ((((("critically ill"[Title/Abstract]) OR "critical illness"[Title/Abstract]) OR ICU[Title/Abstract]) OR "intensive care unit"[Title/Abstract]) OR "critical care"[Title/Abstract]))
#19	((randomized controlled trial[pt] OR controlled clinical trial[pt] OR randomized[tiab] OR placebo[tiab] OR "clinical trials as topic"[MeSH Terms:noexp] OR randomly[tiab] OR trial[ti] NOT ("animals"[MeSH Terms] NOT "humans"[MeSH Terms])) AND Randomized Controlled Trial[ptyp])
#20	#17 AND #18 AND #19
#21	#17 AND #18 AND #19 NOT (1879/1/1[PDAT] :2019/3/31[PDAT])

CENTRAL search strategy（November 11, 2022）

#1	("critical care"):ti,ab,kw (Word variations have been searched)
#2	("critically ill"):ti,ab,kw (Word variations have been searched)
#3	MeSH descriptor: [Intensive Care Units] explode all trees
#4	MeSH descriptor: [Critical Illness] explode all trees
#5	#1 OR #2 OR #3 or #4
#6	MeSH descriptor: [Physical Therapy Modalities] explode all trees
#7	MeSH descriptor: [Occupational Therapy] explode all trees
#8	MeSH descriptor: [Early Ambulation] explode all trees
#9	(mobilizat*):ti,ab,kw

#10	(mobilisat*):ti,ab,kw
#11	(mobility):ti,ab,kw (Word variations have been searched)
#12	(exercis*):ti,ab,kw (Word variations have been searched)
#13	(pregait):ti,ab,kw
#14	(pre-gait):ti,ab,kw
#15	(walk*):ti,ab,kw (Word variations have been searched)
#16	(physiotherap*):ti,ab,kw (Word variations have been searched)
#17	(ambulation):ti,ab,kw (Word variations have been searched)
#18	("physical therapy"):ti,ab,kw (Word variations have been searched)
#19	#6 OR #7 OR #8 OR #9 OR #10 OR #11 OR #12 OR #13 OR #14 OR #15 OR #16 OR #17 OR #18
#20	#5 AND #19 with Cochrane Library publication date Between Apr 2019 and Nov 2022

Ichushi-Web search strategy（November 8, 2022）

#1	((危篤/TH or 危篤/TA)) and (PT=会議録除く)
#2	((クリティカルケア/TH or クリティカルケア/TA)) and (PT=会議録除く)
#3	((ICU/TH or ICU/TA)) and (PT=会議録除く)
#4	((リハビリテーション/TH or リハビリテーション/TA)) and (PT=会議録除く)
#5	((早期離床/TH or 早期離床/TA)) and (PT=会議録除く)
#6	((ランダム化比較試験/TH or 準ランダム化比較試験/TH or ランダム化/AL or 無作為化/AL or 比較試験/AL or 臨床試験/AL or プラセボ/AL or 対照/AL or コントロール/AL or 臨床研究/AL)) and (PT=会議録除く)
#7	#1 or #2 or #3
#8	#4 or #5
#9	#6 and #7 and #8
#10	(#9) and (DT=2019:2022)
