# Supplementary material for: Effects of Mobilization within 72 h of ICU Admission in Critically Ill Patients: An Updated Systematic Review and Meta-Analysis of Randomized Controlled Trials
Source: J Clin Med. 2023 Sep 11;12(18):5888. doi: 10.3390/jcm12185888 (PMC10531519; doi:10.3390/jcm12185888)
Supplement: Supplementary file 1 [file jcm-12-05888-s001.zip › Supplementary Table S3.pdf]

Table S3. Studies excluded in full-text screening

Study	Reason for exclusion
Chan CK et al. American journal of geriatric psychiatry, 2019, 27(3)	Unclear whether rehabilitation interventions are implemented earlier in the intervention group than in the control group
da Rosa Pinheiro DR et al. NeuroRehabilitation. 2021, 48(1)	Intervention is use of an ergometer
Dong Z et al. BMC Pulm Med. 2021 Mar 29;21(1)	Intervention is initiated 72 hours after ICU admission
Hualian W et al. Zhonghua Wei Zhong Bing Ji Jiu Yi Xue. 2021 Nov;33(11)	Different language
Kwakman RCH et al. Crit Care . 2022 Jun;69	Unclear whether rehabilitation interventions are implemented earlier in the intervention group than in the control group
Liu D et al. Nan Fang Yi Ke Da Xue Xue Bao. 2019 Nov 30;39(11)	Different language
Nydahl P et al. Nurs Crit Care . 2022 Jul;27(4)	Intervention is nighttime rehabilitation
Pinkaew D et al. Indian journal of public health research and development, 2020, 11(8)	Intervention is initiated 72 hours after ICU admission
Rahiminezhad E et al. BMC Sports Sci Med Rehabil. 2022 May 26;14(1)	Intervention is range of motion
Rezvani H et al.Crit Care Nurs Q. 2022 Jan-Mar;45(1)	Intervention is initiated 2 hours after ICU admission
Scaillet V et al. Annals of intensive care, 2019, 9	Unclear whether rehabilitation interventions are implemented earlier in the intervention group than in the control group
Veldema J. Acta Neurol Scand . 2019 Jul;140(1)	Patients have been discharged from the ICU
Wang XP et al. J Nurs Res. 2022 Aug 1;30(4)	Intervention is not physical rehabilitation
Wang Z et al. Zhonghua Wei Zhong Bing Ji Jiu Yi Xue. 2021 Sep;33(9)	Different language
Wollersheim T et al. J Cachexia Sarcopenia Muscle. 2019 Aug;10(4)	Intervention is neuromuscular electrical stimulation
Wu H et al. Zhonghua Wei Zhong Bing Ji Jiu Yi Xue. 2021 Nov;33(11)	Different language
Ximenes Carvalho MT et al. Fisioterapia e pesquisa, 2019, 26(3)	Intervention is passive use of an ergometer
Yu H. L et al. Neurol Res . 2019 Sep;41(9)	Unclear whether rehabilitation interventions are implemented earlier in the intervention group than in the control group
Yu T et al. Front Med (Lausanne) . 2022 Jun 9	Unclear whether rehabilitation interventions are implemented earlier in the intervention group than in the control group
Zhengguang W et al. Zhonghua Wei Zhong Bing Ji Jiu Yi Xue. 2021 Sep;33(9)	Different language
Dong Z et al. Int Heart J. 2016;57(2)	Intervention is initiated 72 hours after ICU admission
P Maffei et al. Arch Phys Med	Intervention is initiated 72 hours after ICU admission

Rehabil. 2017 Aug;98(8)	
Moss M et al. Am J Respir Crit Care Med. 2016 May 15;193(10)	Intervention is initiated 72 hours after ICU admission
S Patman et al. Aust J Physiother. 2001;47(1)	Intervention is initiated 72 hours after ICU admission
Abizanda P et al. Maturitas. 2011 Jul;69(3)	Patients in recovery are eligible
Alvarez E et al. J Crit Care. 2017 Feb;37	Unclear whether rehabilitation interventions are implemented earlier in the intervention group than in the control group
Amundadottir O. R et al. European Journal of Physiotherapy, 2021;23	Difference between the intervention and control groups is the number of rehabilitations
Bissett B. M et al. BMJ Open. 2012 Mar 2;2(2)	Not a randomized controlled trial
Boden I et al. BMJ. 2018 Jan 24;360	Patients undergoing scheduled surgery are eligible
Boden I et al. J Trauma Acute Care Surg. 2022 Jun 1;92(6)	Patients are eligible after the start of rehabilitation
Borges D. L et al. J Phys Act Health. 2016 Sep;13(9)	Intervention is use of an ergometer
Brummel N. E et al. Intensive Care Med. 2014 Mar;40(3)	Difference between the intervention and control groups is the number of rehabilitations
Brummel N. E et al. Phys Ther. 2012 Dec;92(12)	Intervention is rehabilitation aimed at cognitive function
de Azevedo J. R. A et al. BMC Anesthesiol. 2021 Nov 13;21(1)	Patients staying in the ICU for > 3 days are eligible
Denehy L et al. Crit Care. 2013 Jul 24;17(4)	Patients staying in the ICU for > 5 days are eligible
Doiron K. A et al. Cochrane Database Syst Rev. 2018 Mar 27;3(3)	Not a randomized controlled trial
dos Santos F. V et al. Physiother Theory Pract. 2020 May;36(5)	Unclear whether rehabilitation interventions are implemented earlier in the intervention group than in the control group
Eggmann, S et al. Trials. 2016 Aug 15;17:403	Not a randomized controlled trial
Fink E. L et al. Pediatr Crit Care Med. 2019 Jun;20(6)	Pediatric patients are eligible
Hickmann, C. E et al. Crit Care Med. 2018 Sep;46(9)	Difference between the intervention and control groups is the number of rehabilitations
Kayambu G et al. BMC Anesthesiol. 2011 Oct 31;11:21	Not a randomized controlled trial
Maffei P et al. Arch Phys Med Rehabil. 2017 Aug;98(8)	Difference between the intervention and control groups is the number of rehabilitations
Nydahl P et al. Nurs Crit Care. 2020 Nov;25(6)	Not a randomized controlled trial
Sarfati C et al. J Crit Care. 2018 Aug;46	Patients staying in the ICU for > 3 days are eligible
Sawada Y et al. Am J Crit Care. 2018 Mar;27(2)	Not a randomized controlled trial
Wright S. E et al. Thorax. 2018 Mar;73(3)	Difference between intervention and control groups is rehabilitation duration
Wu T et al. J Thorac Dis. 2022 Apr;14(4)	Intervention is an extubation program

Camila M D et al. Rev Bras Ter Intensiva. 2012 Jun;24(2)	Not a randomized controlled trial
Ze-Hua D et al. World J Emerg Med. 2014;5(1)	Difference between the intervention and control groups is the number of rehabilitations
TEAM Study Investigators and the ANZICS Clinical Trials Group. N Engl J Med. 2022 Nov 10;387(19)	Difference between the intervention and control groups is intensity of rehabilitation
